# Attitudes towards Olive Oil Usage, Domestic Storage, and Knowledge of Quality: A Consumers’ Survey in Greece

**DOI:** 10.3390/nu13113709

**Published:** 2021-10-21

**Authors:** Georgios Marakis, Fragiskos Gaitis, Spyridoula Mila, Dimitra Papadimitriou, Eirini Tsigarida, Zoe Mousia, Aggeliki Karpouza, Emmanuella Magriplis, Antonios Zampelas

**Affiliations:** 1 Hellenic Food Authority, Kifisias 124 & Iatridou 2, 11526 Athens, Greece; gmarakis@efet.gr (G.M.); fgaitis@efet.gr (F.G.); spyridoulamila@yahoo.gr (S.M.); dpapadimitriou@efet.gr (D.P.); etsigarida@efet.gr (E.T.); zmousia@efet.gr (Z.M.); akarpouza@efet.gr (A.K.); 2 Department of Food Science and Human Nutrition, Agricultural University of Athens, Iera Odos 75, 11855 Athens, Greece; emagriplis@aua.gr

**Keywords:** olive oil, consumer survey, knowledge, safety, polychoric analysis

## Abstract

Consumption of unbranded olive oil obtained in bulk has previously been reported to be very high in Greece, underlining the need to investigate knowledge regarding its health attributes and storage practices, two areas that can affect oil quality. This study aimed to investigate Greek consumers’ use and choice of olive oil, their knowledge about its quality, as well as domestic storage practices of olive oil. A cross-sectional survey was conducted in a representative sample of 857 Greek households that consume olive oil, using a previously validated questionnaire. Most participating households use olive oil produced by themselves or by their extended family or friends (60.3%), and only 27.4% purchase branded olive oil, while 57% reported using extra virgin olive oil (EVOO). Only 38.4% of the respondents reported optimal domestic storage practices to maintain olive oil quality, with a significant greater percentage of non-producers group compared to olive oil producers. In all areas of Greece, the higher the knowledge of olive oil quality, the higher the probability of consumers selecting EVOO and perceiving olive oil price as low. The present survey highlights the need to heighten consumers’ knowledge of olive oil attributes and correct storage practices and awareness about branded EVOO and its superior quality.

## 1. Introduction

Olive oil has been a key staple of the Mediterranean diet since antiquity [1], and research has shown protective effects against cardiovascular diseases and other chronic and degenerative health problems [2,3]. These beneficial health effects are mainly associated with its fatty acid composition—the high content of monounsaturated fatty acids, with oleic acid concentration being predominant—and its numerous bioactive phytochemicals, represented mainly by tocopherols and phenolic compounds, such as oleuropein, hydroxytyrosol, oleocanthal, and oleasin [3,4,5]. These attributes of olive oil that have been linked to its health effects are highly affected by production method, processing, packaging, usage, and storage. The latter two are completely consumer dependent, while packaging preference could also be regarded as another aspect that depends on consumers’ knowledge/awareness and culture. Therefore, consumers’ olive oil knowledge of quality and attitudes towards its usage and domestic storage are important areas of evaluation and research focus, especially in large olive oil markets.

Greece is among the three largest producers in the European Union (EU), along with Spain and Italy [6,7,8], and has one of the largest annual per capita consumption in the world [8]. Approximately one-third of the rural Greek population has previously been reported to be occupied with olive cultivation [9], while many households settled in large urban areas possess and cultivate olive groves [10]. Globally, the olive oil market has evolved from a traditional bulk to a more customized market where olive oil is perceived as a food specialty, like other high-quality products [11], and provides a means to understand consumer behavior. There is no main attribute regarding consumer preference of olive oil [12]. Krystallis and Ness [13], for example, reported that the most valued attributes of olive oil among Greek consumers were geographical origin and organic production, with price being among the least valued. A recent survey [11] reported that Italian consumers had similarly a positive preference for geographical origin attribute, but, on the other hand, the organic attribute was not highly valued. Other attributes, such as oil color [14] and low-acidity [15] as well as attributes such as market leader brand, sustainability, and supply chain ethics [11], have also been reported as important olive oil preference attributes. In Greece, however, previous studies have shown that a large percentage of Greek households continue to consume unbranded bulk olive oil obtained from friends, family, or own production compared to branded products [16,17]. In countries such as Portugal, where olive oil consumers have been shown to exhibit a high degree of ethnocentric behavior [12], such a tendency has not been reported to result or be associated with a preference of unbranded olive oil obtained in bulk. This large difference in Greek consumers compared to other countries, which prefer branded small packages, is an important area of investigation since the quality of unbranded oil and factors previously stated as affecting its attributes cannot always be tested. Therefore, knowledge with regards to olive oil quality is of great importance.

Several studies have evaluated consumers’ subjective knowledge of olive oil and on methods consumers use to choose/purchase it [1,16,18]. The study by Santosa and colleagues [18] pointed that even though California consumers perceive olive oil as a “healthy” food, most were unaware of the bioactive components of olive oil and the link between some of these components with the sensory properties of bitterness, astringency, and pungency. This is in accordance with another study conducted in Spain and the U.S., which showed that U.S. consumers liked bland, refined olive oil as opposed to Spanish consumers that preferred more pungent taste and showed that Spanish consumers were more aware about olive oil quality than those in the U.S., underlining olive oil knowledge of health attributes to preference [1]. Although quality is a multidimensional aspect, consumers’ knowledge of attributes that differentiate different types of olive oil are important, particularly those that are easily detectable through taste and flavor. In a recent Italian study [11], even though 73% of the participants stated that they knew the characteristics of EVOO, only just above 50% were able to correctly recognize those characteristics that distinguish this product. In general, previous surveys in Greece have described consumers’ preferences, attitudes, and perceptions about olive oil characteristics [19]; consumers’ perception regarding olive oil authenticity [20]; and the economic and socio-spatial attributes that affect consumers’ choices on purchasing olive oil from the supermarket or from a friend/relative or consuming their own production [10]. Overall, many of the attributes that guide consumer preference and purchase are conveyed to the consumers through the label information and the certification logos [11]. When olive oil is obtained in bulk (unbranded), as is the case in Greece up to recent studies, consumers need to rely on their knowledge regarding choice and its subsequent domestic storage. In Greece, such data regarding consumers’ knowledge on the quality of olive oil as well as on consumers’ behavior with respect to domestic storage practices are limited, thus distinguishing our survey from previous ones.

The aim of this study was to assess olive oil purchase, usage, storage behavior, and knowledge related to olive oil quality of Greek consumers. In addition, we aimed to assess how knowledge score is associated with various consumer characteristics and behavior.

## 2. Materials and Methods

### 2.1. Participants

This survey was conducted in Greek households consuming olive oil through telephone interviews by a professional market research company based in Athens (“Hellenic Research House” (HRH)) using a stratified random sampling. Sample allocation was performed based on the number of households by region, according to sample list extracted from telephone directory, based on Census 2011. Survey participants were randomly selected, one from each household, and were eligible if they were adults (i.e., >18 years old) and reported of being responsible for food purchase or food preparation in the household.

**Figure 1:**
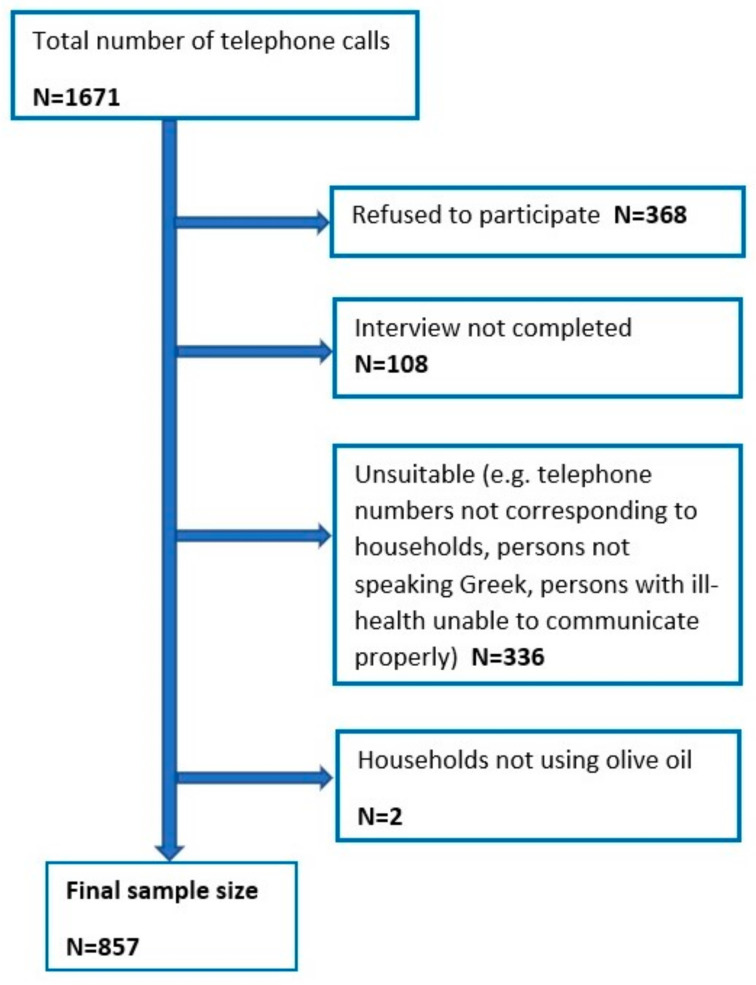
Flow chart of sampling procedure.

The sample size was calculated aiming an 80% study power at the 5% level, accounting for type of study and the aim. A design effect of 1.5 and 50% prevalence (0.5) of adequate knowledge was estimated, as recommended when other relevant national data are unavailable. This resulted in 576 individuals, which was divided by a projected 70% response rate. A total of 823 individuals were required. The final number of households that were selected to participate in the survey was 857. The sampling procedure is depicted in Figure 1.

Data collection was conducted in May 2020 by trained personnel using Computer-Assisted Telephone Interviewing (CATI). The mean time required per respondent to complete the survey was 18 min.

Participation in this survey was voluntary and anonymous, and they were made aware that they could withdraw at any time from the study. All subjects gave verbally their informed consent prior to their participation. The study was conducted in strict accordance with the ICC/ESOMAR Code on Market and Social Research and in accordance with the guidelines given in the Declaration of Helsinki. The protocol was approved by the Management Board of the Hellenic Food Authority (Decision No: 303/19-07-2019).

### 2.2. Questionnaire

Questions covered basic personal and sociodemographic characteristics, nationality and family size, olive oil cost perception, type of oil consumed, type of oil bought (“packaging”), knowledge on olive oil quality attributes, and storage practices. These were mostly either single- or multiple-choice/check–all-that-apply and close-ended. Likert scale was not used for responses that required subjectivity since specific responses were needed. Specifically, data were acquired for sex, age category, area of residence, level of education, type of profession, and olive oil price perception (very low to very high). Age was initially categorized in 4 groups (18–30; 31–50; 51–65; and ≥65) but was regrouped in 3 for further analysis (18–50).

Level of education was categorized as “basic-elementary”, “high-school”, and “higher”. A list of 17 choices was initially included in the questionnaire for profession-type assessment (the question about the participant’s job was open-ended; details of answer choices are not included in the Supplementary Materials). However, this was grouped into 8 groups at the analysis stage based on level of expertise and social determinants; “unemployed” (including those responding students since all individuals are adults), “housework”, “farmer”, “blue collar workers”, “white collar workers”, “freelancers”, “company owner-rentier”, and “teacher/professor”. As per nationality, only 15 (1.7%) respondents were foreigners and were therefore not analyzed separately.

Income-associated questions were posed at the end of the survey in order to reduce the risk of drop-out. Data on monthly income were not included in the analysis of this study due to observed high recall bias. Money spent on food per month accounting for number of household members was used as a proxy measure for income status.

The type of olive oil consumed and the usual as well as the occasional use of olive oil in salads and in food preparation by the study participants were identified without an attempt to quantify the amount consumed. Participants were asked whether their family or their relatives produce olive oil (variable olive oil production—yes/no) and whether in their household the olive oil is obtained/purchased as bottled (branded) or in bulk (unbranded) or both. If they purchase branded olive oil, participants were asked to identify those characteristics indicated in the package that are important to them (such as acidity, Protected Designation of Origin (PDO)/Protected Geographical Indication (PGI) claims, and price).

### 2.3. Questionnaire Internal Validation

The questionnaire was initially developed by scientists at the Hellenic Food Authority and was then distributed for comments and corrections to academic olive oil experts. It was tested for face and content validity by assessing the phrasing and clarity of instructions.

Prior to data collection commencement, the questionnaire’s reliability over time was tested by administering to a small group of volunteers/test-takers (*n* = 53) on two different occasions, approximately 10 days apart. The inter-subject coefficient of variance (CV) was 13.96%, and the intra-subject coefficient of variance (CV) was 4.61%. Chi-square tests showed that the answers to the questions were not significantly different between the two interviews (*p* > 0.05). For the estimation of Pearson’s correlation coefficient, the questionnaire was divided into six sections, based on topic covered (Supplementary Materials), and the reliability for each section was calculated. The correlation coefficients ranged from 0.65 to 0.85. The overall Pearson’s correlation coefficient—which can fall between −1 (complete negative correlation), 0.00 (no correlation), and 1.00 (perfect positive correlation)—was 0.76 (high) for this questionnaire, which is regarded as “acceptable”.

A total of 18 questions, with 4–6 subcategories that can be chosen in 11 of these, were analyzed for the purpose of this study. The main purpose was to assess knowledge of olive oil quality and domestic storage knowledge (details provided in next Section 2.4). Internal consistency of the specific questions was assessed using Cronbach alpha statistic. This test assesses the reliability of a summative rating. In total, Cronbach alpha for the complete questionnaire was 0.72 (average inter-item covariance 0.02); for nutrition knowledge and storage score questions, this was 0.71 (average inter-item covariance 0.01). A value above 0.7 is considered reliable, considering that multiple questions were used to measure overall knowledge and storage practices.

### 2.4. Knowledge and Domestic Storage Practices Scores

Knowledge on olive oil quality was assessed using seven questions (Questions 5–8 and 10–12 in Supplementary Materials). These were selected based on experts and extra virgin olive oil attributes important for health [21]. A Likert scale for perceived knowledge was not provided since the aim was the evaluation of actual knowledge. An “olive oil quality knowledge score” was calculated by giving one point for each correct answer. Since in some questions more than one answers were correct, the score ranged from 0–12. The highest score (i.e., 12) would be assigned to those indicating: “olive oil is of superior quality if it has low acidity (1), bitter taste with spicy notes (2), derived by cold mechanical extraction (3), and is organic (4)”; “olive oil can have bitter or spicy notes because it is rich in phenolic compounds with antioxidant effects (5)”; “an olive oil is extra virgin when its acidity less than 0.8% (5)”; “extra virgin olive oil is superior to refined olive oil with regard to its quality characteristics (7)”; “olive oil is suitable raw in salads and dishes (8), for cooking at low temperatures (9), and for frying at temperatures up to 190 °C (10)”; “olive oil is best consumed immediately after its extraction (11)”; and “in comparison to other types of vegetable oils, olive oil is more nutritious/healthier (12)”. The correct answers were based on information from the European legislation on olive oil (Regulation (EU) 2016/2095) [22] and relevant scientific articles, e.g., [3,6]. Among the attributes that constituted the score, the superiority of organic EVOO could be debated as previously suggested [23] since many parameters must be controlled to detect whether the nutritional quality of organic olives is higher than the conventional ones. However, it was decided to be included as an attribute related to superior quality. This was based on evidence regarding the larger concentration of total phenols and other bioactive compounds in the organic oils compared to the conventional ones and the lack of use of synthetic fertilizers, pesticides, and growth regulators of olive trees grown organically [24,25].

Questions about domestic storage practices (Questions 13 and 14 in Supplementary Materials) were posed to the respondents with the aim to assess potential quality degradation and safety issues related to the suitability of the contact material of the container [19]. For the purpose of analyses, the two questions were grouped together, and we regarded best storage conditions as “in a cupboard without sunlight and cool” and ideal containers as “bottles as purchased from retail shops” or “green- or brown-colored glass bottle”. These practices are important to maintain chemical properties highly linked to health benefits as well as taste [26].

### 2.5. Statistical Analyses

All categorical variables are summarized with frequencies and percentages. Based on the study aim, data were stratified by olive oil production. All participants that responded positively in questions about whether their family or their relatives produce olive oil (either/or) were included in “production”. Chi-square test was used to assess statistical significance between groups at the 5% level (a = 5%). Total knowledge score was depicted as mean (sd) since it was normally distributed and was then made categorical based on the population’s median for further analysis. This was done since the knowledge score has not been previously validated, and there was inadequate statistical power for more detailed percentile analysis. Minimally adjusted logistic regression (for age category and sex) was used in a preliminary analysis in total and then stratified by olive oil production. A polychoric analysis, which is a principal component analysis for categorical variables, was conducted to identify main components that characterize the variation in specific socioeconomic and family-related variables, found to be associated with knowledge score. Polychoric correlations assume the variables are ordered measurements of an underlying continuum, based on maximum likelihood, and can range from −1 to 1 inclusive and measure the strength and direction of the association between two variables. All components are uncorrelated with one another. Interpretation of the components is based on findings which variables are most strongly correlated with each component, based on their loadings (loadings >0.3 in absolute terms add to the component, >0.5 is deemed overall important). The variables that are correlated are those that the specific component is explained by. Based on the components identified, a multivariable logistic regression was performed to account for all possible confounders while limiting type 2 error. STATA 14.0 (StataCorp, Texas Ltd., College Station, TX, USA) statistical package was used for the analysis.

## 3. Results

A summary of the main characteristics of study population, including sociodemographic data stratified by olive oil production, is depicted in Table 1. Stratification was performed since 60.3% of participating households (including their extended family or friends) produce olive oil. Significant differences in total sample were seen for area of residence, total household members (*p* < 0.001), and monthly grocery budget per household member while accounting for household members for the later (*p* = 0.03). Additionally, a statistically significant difference was observed with respect to the type of olive oil purchased (branded vs. non-branded) between those who produce olive oil and those who do not (*p* < 0.001), but no significant difference was observed in relation to the quality of olive oil consumed (extra virgin vs. other types) (*p* = 0.165). In addition, those whose family/relatives produce olive oil seem to be significantly less concerned about olive oil fraud (*p* < 0.001) and significantly more knowledgeable about olive oil quality characteristics (*p* < 0.001) although not for domestic storage practices (*p* = 0.026). No statistically significant difference was observed regarding their concern about fraudulent practices in table olives (*p* = 0.09). For further analysis, age group was regrouped into three instead of four categories to increase power of statistical analysis since only 62 individuals aged 18–30 years were the main household food providers and since no significant between group differences were observed in preliminary analysis.

**Figure 2:**
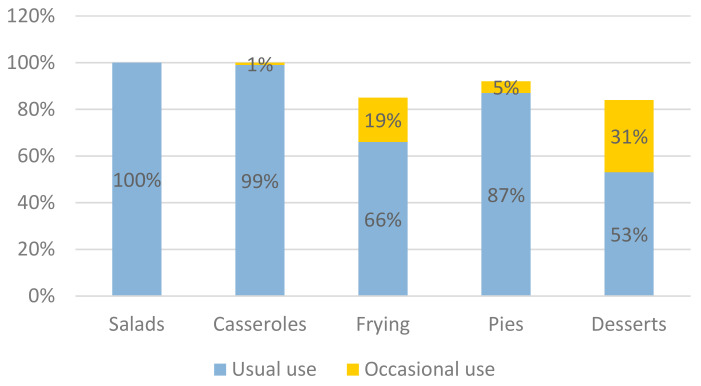
Usual and occasional use of olive oil in food preparation in Greek households.

Survey results also showed that olive oil use during food preparation in Greek households is very high (Figure 2). All households use exclusively olive oil in salads and almost exclusively in casserole dishes, whereas 85% of households use olive oil for frying, 92% for making pies, and 84% for preparing desserts usually or occasionally. When frying, 28% of the participants also reported using exclusively or occasionally other types of vegetable oil, such as sunflower oil or corn oil, while for the preparation of desserts, 24% of the respondents also reported the use of butter. Margarine seems to be less preferred in food preparation, mainly used by 8% of the participants for making desserts.

Only 27.4% of the participating households purchase branded olive oil (Table 1). Among those, 63% are loyal to a specific brand. Price was the most frequent attribute reported (by 54% of buyers of branded olive oil) that influences the purchase of branded olive oil. Other selection criteria that appeared to be important for branded products were the acidity value of olive oil (44%), its geographical indication (39%), an indication of Protected Designation of Origin (PDO) (30%), the material of its container (24%), and any health claims appearing on the label (17%) (data not shown).

With respect to consumers’ knowledge on olive oil characteristics, the acidity value, which is one of the criteria to differentiate extra virgin olive oil from other types of olive oil, was pinpointed by more than half of the study participants. In particular, 60% of the sample knew that a high-quality olive oil should have low acidity value, while more specifically, 54% of all respondents knew that an olive oil is extra virgin when its acidity value is below 0.8%. It should be noted that acidity value is typically stated on the label of branded olive oil products or provided to producers following analysis at the premises of olive mills immediately after the oil extraction since it is an attribute that cannot be determined by our senses.

Nearly eight out of ten participants (78%) knew that extra virgin olive oil is of higher quality than refined olive oil, and 43% reported that they are able to distinguish between extra virgin and refined olive oil based on organoleptic characteristics. However, when participants were asked to indicate why an olive oil can have bitter or spicy notes, only 19% correctly answered that the polyphenols in EVOO are responsible for this taste and flavor. A total mean score of 6.4 (1.9) was calculated for the study population, with significant group differences: 6.6 (2.0) in those grouped in oil production and 5.9 (1.9) in non-producers.

Regarding domestic storage practices of olive oil, 38.4% of the respondents reported storing olive oil in dark and cool place (away from sunlight) and in containers either as sold (for branded products) or in dark-colored glass containers. However, most respondents (61%) keep the olive oil in large metal/tin containers, and 6% stated that they transfer the olive oil from tin/metal large containers to plastic bottles that had previously been used for other types of liquid, such as water or fizzy drinks. In addition, 5% of the participants keep olive oil on the kitchen bench next to kitchen stoves and not in a dark and cool place. Only 38.4%, therefore, of the sampled population acquired a full score (=1) for domestic storage practices, with significantly more non-oil producers achieving full score (42.9 % vs. 35.9%, respectively; *p* = 0.026).

A minimally adjusted logistic regression analysis (for sex and age) of Consumer Knowledge Score was performed to investigate its association with the type of oil consumed and the domestic storage practices in total population and by olive oil production status. As presented in Table 2, higher-knowledge consumers were 1.86 times more likely to use EVOO compared to lower-knowledge consumers (95% CI: 1.393–2.475). In addition, those with higher knowledge score were more likely to have concerns about fraudulent practices in table olives. Furthermore, consumers with higher knowledge score were 50% less likely to use branded olive oil (32% to 64%) and 35% less likely (23% to 44%) to perceive olive oil price as high.

Significant associations were also observed in relation to the place of residence, the profession type, and the level of education. Specifically, consumers with higher educational level, better profession, as well as not residing in Attica were more likely to achieve a higher knowledge score of olive oil quality.

Based on these results, we aimed to identify how the characteristics associated with knowledge score are associated and explain the population variance, hence generating consumer profiles. To create these, polychoric analysis was selected as the most suitable statistical method (Table 3). For this statistical analysis, the age groups “18–30 years” and “31–50 years” were grouped together since the sample of individuals 18–30 years old was small for further analysis to be performed. Additionally, the number of children in household was categorized as binary (no children and yes children in household). This question did not define whether respondents had children but if a child (<18 years old) still lived in the household with them.

Two profiles of the consumers were finally selected for further investigation. The first was the one that explained 36.6% of the sample variation and was characterized by adults aged 18–50 years with higher educational level and with ≥3 household members, including children (Component 1). The second profile explained 22.6% of the variation and was characterized by household-chore-performing females of lower education (Component 2).

A multivariable logistic regression, by consumer profile, was used to investigate the probability of having higher (or lower) Consumer Knowledge Score with the type of oil consumed and domestic storage practices. As shown in Table 4, profile 1 was associated with higher odds of knowledge score, whereas profile 2 with lower.

For both profiles, knowledge score was positively associated with the use of EVOO and negatively associated with the purchase of branded products. Those living in Crete and the islands (both profiles) had higher knowledge score compared to those in Attica (73 times in profile 1 and 81 times in profile 2), whereas more individuals living in northern Greece (both profiles) were less knowledgeable about olive oil quality (40% in profile 1 and 38% in profile 2).

**Figure 3:**
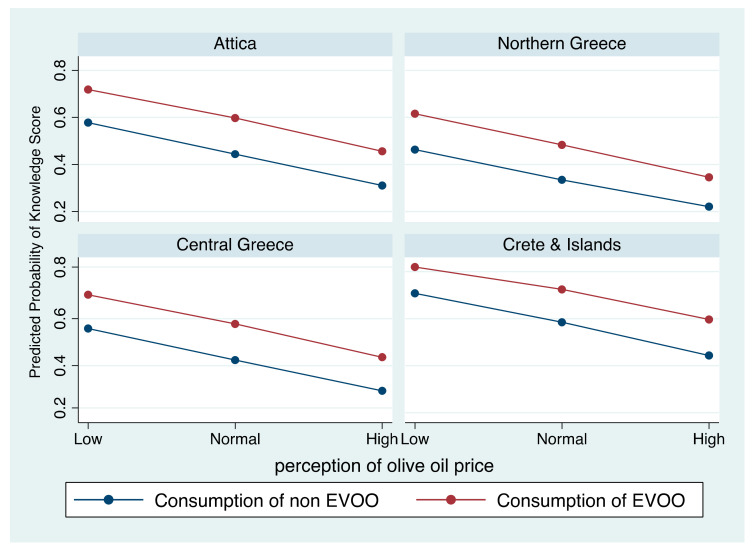
Adjusted Predicted Probability of extra virgin olive oil consumption according to knowledge score and price perception by area.

Significant indicator variables from the adjusted logistic regression were further evaluated to assess their relation in relation to Knowledge score. Figure 3 depicts the predicted proportion of individuals that use EVOO based on price perception according to knowledge score in each area assessed, following a multivariable logistic regression (as shown in Table 4). Specifically, in all areas, the higher the knowledge score, the higher the probability that consumers select EVOO and perceive olive oil price as low.

The higher probability of EVOO consumption with knowledge score was observed in Crete and Islands (≈82%). However, in this area, more consumers scored a low knowledge score and perceived olive oil price as high (≈60% compared to 34% in Northern Greece and ≈45% in Attica and Central Greece). Attica and Central Greece had overall almost the same probabilities, and Northern Greece had the lowest knowledge score and probability of EVOO consumption.

## 4. Discussion

This survey confirmed that although most Greek consumers use olive oil irrespective of brand, not all use EVOO, which is of superior value and has potential to promote multifunctional and sustainable agricultural models [11]. In addition, a high percentage of households sampled scored low in knowledge pertaining to healthy olive oil attributes, and 61.6% of the population stored it correctly. This underlines potential concern areas since not only is olive oil bought in bulk, but knowledge on quality and storage methods can decrease the health effectiveness despite the almost sole use of olive oil in Greek households. Although many achieved a good knowledge score, domestic storage practices were not optimal, especially among the group consuming olive oil produced by family or relatives. This is an important finding since domestic storage practices is key to maintaining olive oil quality.

Study findings on olive oil preference are in accordance with previous data from Greece [13] and studies conducted in other Southern European Countries, such as Spain [1]. It is also in line with Greece’s national dietary guidelines for adults that recommend the use of olive oil as the main source of fat [27]. Interestingly though, only 66% reported the use of olive oil as the main type of oil for frying, and an additional 19% use it occasionally when frying; a percentage like that was reported (69.5%) in a previous, nationally representative study conducted by the Hellenic Food Authority [28]. As olive oil is more resistant to deterioration when frying if reused compared to other types of vegetable oils such as sunflower oil [29], this needs to be better communicated to reach more Greek consumers.

Extra virgin olive oil (EVOO) is regarded as the highest quality olive oil due to its organoleptic characteristics, its stability, and its high content of numerous bioactive compounds conferring significant health benefits. However, only 57% of Greek households reported its use in this survey, which is less than that (i.e., 70%) reported 25 years ago by Siskos and coworkers [30] and also less than that reported in recent surveys among Italian consumers (i.e., over 90%) [11] and Spanish consumers (78.2%) [31]. This unexpected finding could be attributed on one hand to the economic crisis since EVOO is more expensive compared to other types of olive oil and, on the other hand, to differences in knowledge and understanding of the terminology that is used to classify different types of olive oil. The high percentage of those preferring unbranded olive oil may also partly explain this finding since unbranded olive oil typically does not bear such indication on its container. It is noteworthy though that there was no significant difference observed on the type of olive oil used between those whose family/relatives produce olive oil and those who are not olive oil producers. However, independent of the variables included in the analyses, better knowledge was associated with higher consumption of EVOO.

Nearly three quarters of the participating households stated that they obtain olive oil in bulk from family and friends. The relatively small market share of branded olive oil in Greece has also been pointed out in recent articles from Greece [16,32]. This could be partly explained by the fact that 60% of the participating households are producers themselves or their extended family are, and hence, many would be willing to support family/friends and/or local economy. Furthermore, the lack of efficient branding and promotion strategies in olive oil, as identified previously by Sandalidou and coworkers [9] and Baziana and Tzimitra-Kalogianni [32], the lower price of olive oil sold in big quantities, and the fact that consumers generally value positively the geographical origin of olive oil [33,34] may also account for the high preference of unbranded olive oil purchased in bulk. However, the quality or the safety of olive oil/table olives obtained in bulk (unbranded) cannot always be assured and/or easily inspected by authorities.

Regarding packaged (branded) olive oil, Vlontzos and Duquenne previously reported that its preference is related to socio-spatial characteristics of households, such as age, level of education, and household’s proximity to olive oil production as well as place of origin, and found no association with household’s size or family income [10]. In our survey, the most important attribute among branded olive oil purchasers was price followed by acidity value and geographical location/origin. Overall geographical location is generally known for influencing the perceived quality of olive oil [14]. The price factor is highly variable, with earlier research from Greece [17,30] showing that the price of EVOO had little influence as selection criterion, but our findings are post economic crisis and can explain the results. In line with our results and hypothesis of economic crisis research after 2010, Perez and collaborators [34] reported that price was the most important attribute for the purchase decision, driving 64% of the final choice of olive oil. Similar findings have also been observed in a study with French and Tunisian consumers [35] and in an Italian survey [36]. Interestingly, although price was reported to be important, only about a third of the whole survey population considered the price of olive oil as high/very high, a factor that highlights the perceived economic value of this food item. This observation is reinforced by our finding that in all areas of Greece the higher the knowledge score, the higher the probability that consumers select EVOO and perceive olive oil price low, presumably because those who are aware of its health and nutritional benefits would value greatly this type of olive oil. Educational initiatives, therefore, could promote better understanding and provide scientifically sound information on how to distinguish between different types of olive oil. For example, if more consumers could become more aware that phenolic compounds are beneficial to health and contribute to the bitter and pungent “picante” attributes, which are distinctive of high-quality EVOOs [35], it is likely that more consumers would seek these organoleptic characteristics and consciously choose such olive oils. Another point that needs to be stressed is that the European Union has included in the list of permitted health claims on foods the claim that “olive oil polyphenols contribute to the protection of blood lipids from oxidative stress”. This claim, when stated on labels, can allow consumers to recognize the highest quality products within the category of EVOO and also increase the purchase of branded olive oil [37].

The knowledge of the respondents regarding olive oil quality characteristics appeared to be better among those whose family are olive oil producers and those with higher educational level and better profession. It is believed that those with higher level of education are better able to seek and interpret any nutrition-related information and, as a result, have a better-quality diet [38]. Polychoric correlation revealed that particularly adults (18–50 years) with higher educational level and with ≥3 household members, including children, were more likely to have better knowledge on olive oil, also reinforcing the notion that marital status tends to be associated with attaching greater importance to olive oil quality [20].

According to data from the Ministry of Rural Development and Food in Greece, almost 80% of olive oil production is centered in three regions: Peloponnese (37%), Crete (30%), and the Ionian Islands (12%). On the one hand, this partly explains the finding that Northern Greece had the lowest knowledge score and probability of EVOO consumption. However, considering that a greater percentage of consumers from Crete and the islands appeared to have low knowledge score, it would be advisable that consumers’ educational programs are implemented on a national scale rather than targeted to specific non-producing olive oil areas of Greece.

The influence of storage conditions and type of packaging container on olive oil quality is well-established, with dark-colored (green or brown) glass containers preserving best its benefits to health and sensorial properties [39]. The material of the container, though, was not picked as one of the most important attributes when choosing branded olive oil in this survey, and those responding correctly were consumers that bought branded olive oil and hence did not have to change containers. Similarly, packaging was reported to be the least important attribute in an olive oil consumer survey in France and Tunisia [35]. Tin box packaging was generally preferred by more than half of the participating households for olive oil in our survey, possibly due to local experience and other culture-related factors. This finding is consistent with a study conducted in Turkish consumers [40]. Nonetheless, large tin containers (such as 17 kg containers, which are frequently used) may contain appreciable amounts of oxygen in the headspace, particularly as the storage time increases, which can facilitate the oxidative rancidity and deterioration of olive oil quality and further minimize health effects. Furthermore, a small proportion of the participants in this survey transfer the olive oil from tin/metal large containers to plastic bottles that had previously been used for other types of liquid with various thickness and composition. This implies lack of awareness regarding safe domestic storage practices since plastic material not intended for oil storage may not be preferred or suitable due to the potential migration of small molecular weight compounds from the bottle to oil and permeability of the wall to gases and vapor, such as oxygen and humidity [41]. The fact that a significant proportion of non-olive oil producers had better domestic storage practices compared to producers can be explained by the fact that more non-producers buy branded olive oil. This means that they may comply with storage practices due to the fact that branded olive oil is sold in appropriate packages and not due to knowledge. Incorrect storage practices did not seem to be influenced by consumer profile or by other attributes, such as olive oil production. Our findings therefore suggest the need to heighten consumer awareness campaigns about best storage practices to preserve the quality of olive oil.

Finally, the study population did not seem to be overly concerned with olive oil adulteration possibly because they have better knowledge of how the olive oil is produced and confidence in the origin and quality standards of the olive oil that the family or close friend gives them. This is desirable since the fear of olive oil adulteration could potentially negatively affect consumers’ behavior and decrease the valuation of the product [20]. Olive oil producers were significantly less concerned about potential olive oil adulteration practices compared to the ones who are not olive oil producers. However, olive oil is a high-value product and consequently vulnerable for adulteration/deliberate mislabeling [19,23]. The violation of the olive oil legislation, which is reported by official controls in Greece, may be attributed on the one hand to economic crime and on the other hand to the indifference, tolerance, and/or ignorance of the consumers. Therefore, targeted consumer awareness policies and actions about olive oil are of utmost significance.

### Limitations of the Study

The data were self-reported, and hence, they may be subject to reporting bias although this is a consumer survey on perception and knowledge. In addition, the type of olive oil used in the households could not be confirmed. Since consumers are often confused because of the complicated terminology used to classify different olive oil types, these results should be treated with caution. The use of real choice study in the future would be advisable to avoid hypothetical bias and confirm the findings.

## 5. Conclusions

This survey highlights areas that need to be attained to regarding consumers’ knowledge, storage, and behavior towards olive oil. Nevertheless, there are some knowledge gaps and malpractices that have been addressed and need to be improved. The use of unbranded in bulk olive oil in Greek households remains high and is significantly associated with olive oil production, but its quality and safety is not ascertained. Therefore, olive oil education programs are necessary since only approximately half of the households with higher educational level and better profession reported using EVOO, and better knowledge was associated with higher EVOO consumption. Future investigations could encompass actual olive oil consumption as well as quality assessment of produced olive oil. Lastly, it may be commendable to include more households to have a representative sample of olive oil attitudes from other ethnicities as well. Educational efforts to promote better understanding of the superiority of extra virgin olive oil are warranted, such as learning to appreciate the bitter and pungent taste of the EVOO.

## Figures and Tables

**Table 1:** Main characteristics of study participants in total (*n* = 857) and by olive oil production.

Participants	Total	Olive Oil Production ^1^		*p*-Value ^2^
		Yes	No	
	857	517 (60.3)	340 (39.7)	
Sex, *n* (% females)	64.2	339 (65.6)	211 (67.1)	0.294
Age, *n* (%)				0.815
18–30 years	62 (7.2)	38 (7.4)	24 (7.1)	
31–50 years	324 (37.8)	189 (36.6)	135 (39.7)	
51–65 years	251 (29.3)	156 (30.2)	95 (27.9)	
>65 years	220 (25.7)	134 (25.9)	86 (25.3)	
Area of residence, *n* (%)				<0.001
Attica	342 (39.9)	200 (38.7)	142 (41.8)	
Northern Greece	218 (25.4)	87 (16.8)	131 (38.5)	
Central Greece	196 (22.9)	142 (27.5)	54 (15.9)	
Crete and Islands	101 (11.8)	88 (17.0)	13 (3.0)	
Professional status				0.094
Housework	154 (18.0)	98 (19.0)	56 (16.5)	
Unemployed	57 (6.6)	34 (6.6)	23 (6.8)	
Farmer	48 (5.6)	39 (7.5)	9 (2.7)	
Blue collar worker	46 (5.4)	27 (5.2)	19 (5.6)	
White collar worker	361 (42.1)	203 (39.3)	158 (46.4)	
Freelancer	87 (10.1)	53 (10.3)	34 (10.0)	
Company Owner-Rentier	40 (4.7)	25 (4.8)	15 (4.4)	
Teacher-Professor	64 (7.5)	38 (7.3)	26 (7.6)	
Educational level, n (%)				0.073
Elementary	107 (12.5)	75 (14.5)	32 (9.4)	
High School	419 (48.9)	251 (48.6)	168 (49.4)	
Higher education	331 (38.6)	191 (36.9)	140 (41.2)	
Household members				0.042
1–2 members	377 (44.0)	213 (41.2)	164 (48.2)	
≥3 members	480 (56.0)	304 (58.8)	176 (51.8)	
Number of children in household ^3^				0.304
None	597 (69.7)	350 (67.7)	247 (72.7)	
1 child	97 (11.3)	62 (12.0)	35 (10.3)	
≥2 children	163 (19.0)	105 (20.3)	58 (17.1)	
Monthly budget for supermarket ^4^				0.03
≤150 euros	342 (14.3)	229 (15.5)	113 (12.4)	
151–200 euros	368 (15.4)	207 (14.0)	161 (17.6)	
201–250 euros	342 (14.3)	227 (15.4)	115 (12.6)	
251–300 euros	368 (15.4)	214 (14.5)	154 (16.9)	
301–400 euros	427 (17.9)	249 (16.9)	178 (19.5)	
≥401 euros	541 (11.7)	349 (23.7)	192 (21.0)	
Type of oil obtained/bought				<0.001
Branded	235 (27.4)	36 (6.7)	199 (58.5)	
Non-branded ^5^	622 (72.6)	481 (93.0)	141 (41.5)	
Type of olive oil consumed				0.165
Extra virgin olive oil	488 (56.9)	283 (54.7)	205 (60.3)	
Virgin olive oil	320 (37.3)	198 (38.3)	122 (35.9)	
Other	6 (0.7)	4 (0.8)	2 (0.6)	
Don’t know	43 (5.0)	32 (6.2)	11 (3.2)	
Oil adulteration concerns ^5^				
(yes/no)	133 (15.5)	33 (6.4)	100 (29.4)	<0.001
Olive adulteration concerns ^6^	273 (31.9)	176 (34.0)	97 (28.5)	0.090
Knowledge Score (0–12), mean (sd)	6.4 (1.9)	6.6 (2.0)	5.9 (1.8)	<0.001
Knowledge score > median, *n* (%)	416 (48.5)	283 (54.7)	133 (39.1)	<0.001
Domestic Storage Practice Score (% with score 1)	329 (38.4)	183 (35.4)	146 (42.9)	0.026

^1^ Olive oil production included all individuals that responded “yes” in questions about whether their family or their relatives produce olive oil (either/or). ^2^ Significance at 5% level; group differences by olive oil production; analyzed using chi-square test. ^3^ Defined as <18 years of age. ^4^ Weighted by number of household members. ^5^ Non-branded includes own production, gift/bought from family/friends, or bought from distributors and olive mills. ^6^ 46 participants reported they have never thought of it; these were also categorized as “no”.

**Table 2:** Minimally adjusted logistic regression (for sex and age) of Consumer Knowledge Score association with type of oil consumed and domestic storage practices in total population and by olive oil production status.

	Total (*n* = 857)	Olive Oil Production	
		Yes (*n* = 517)	No (*n* = 340)
Oil-related variables	OR (95%CI)	OR (95%CI)	OR (95%CI)
Domestic Storage Practices (0–1)	1.17 (0.886–1.546)	1.26 (0.873–1.819)	1.23 (0.784–1.925)
Selection of EVOO compared to virgin	1.86 (1.393–2.475) ***	2.17 (1.494–3.145) ***	1.72 (1.065–2.763) *
Branded vs. Non-Branded	0.50 (0.366–0.679) ***	0.512 (0.255–1.028)	0.70 (0.450–1.100)
Price perception	0.65 (0.555–0.770) ***	0.58 (0.476–0.716) ***	0.96 (0.707–1.296)
Adulteration in olive oil	0.55 (0.810–1.486)	0.86 (0.496–1.504)	0.97 (0.657–1.424)
Adulteration in olives	1.53 (1.141–2.044) **	1.60 (1.100–2.322) *	1.29 (0.794–2.100)
Socioeconomic-related variables	OR (95%CI)	OR (95%CI)	OR (95%CI)
Children in household (yes/no)	1.0 (0.731–1.366)	1.0 (0.675–1.490)	0.91 (0.533–1.547)
Area (baseline Attica)	1.18 (1.035–1.341) *	1.12 (0.964–1.312)	1.09 (0.842–1.423)
Profession type ^1^	1.26 (1.104–1.433) ***	1.28 (1.082–1.512) **	1.30 (1.043–1.623) *
Educational level	1.45 (1.159–1.807) ***	1.56 (1.176–2.076) **	1.46 (0.997–2.128)
Monthly budget for groceries ^2^	1.07 (0.993–1.163)	1.06 (0.955–1.168)	1.10 (0.965–1.262)

Model adjusted for age category and sex; Significance set at 5% level; CI, confidence interval. * *p* < 0.05; ** *p* < 0.01; *** *p* < 0.001. ^1^ profession type was grouped into 4 groups for analysis purposes (Group 1: “housework” and “unemployed”, Group 2: “farmer” and “blue collar worker”, Group 3: “white collar worker”, and Group 4: “freelancer”, “company owner/rentier”, and “teacher/professor”; reference group housework. The analysis was not stratified due to non-significant differences, so final group results are depicted). ^2^ adjusted for number of household members.

**Table 3:** Consumer profile characteristics following polychoric analysis. Principal components (Eigen vectors) (blanks are abs (loading) <3).

	Component 1	Component 2
Eigen Value	2.6	1.6
Proportion of Variance explained	36.6	22.6
KMO	0.65	
Variables included in polychoric analysis		
Profession ^1^	0.205	−0.565
Age category	−0.480	0.019
Educational level	0.340	−0.440
Sex	−0.019	0.592
Household members (number)	0.513	0.258
Number of children in household	0.517	0.254
Monthly budget for groceries	0.292	0.076

^1^ profession type was grouped into 4 groups for analysis purposes (Group 1: “housework” and “unemployed”, Group 2: “farmer” and “blue collar worker”, Group 3: “white collar worker”, and Group 4: “freelancer”, “company owner/rentier”, and “teacher/professor”).

**Table 4:** Multivariable logistic regression of Consumer Knowledge Score association with the type of oil consumed and domestic storage practices in total and by consumer profile.

Knowledge Score	OR (95% CI)	
	**Profile 1**	**Profile 2**
Profile ^1^	1.36 (1.009–1.846) *	0.63 (0.470–0.853) **
Domestic Storage Practices (0–1)	1.26 (0.927–1.723)	1.26 (0.924–1.721)
Selection of EVOO compared to virgin	2.00 (1.466–2.718) ***	1.91 (1.398–2.602) ***
Branded vs. Non-Branded	0.84 (0.555–1.265)	0.81 (0.540–1.226)
Price perception		
Normal vs. Low	0.55 (0.350–0.858) **	0.57 (0.363–0.895) *
High vs. low	0.29 (0.178–0.459) ***	0.31 (0.196–0.504) ***
Area (baseline Attica)		
Northern Greece	0.60 (0.350–0.860) **	0.62 (0.421–0.907) *
Central Greece	0.92 (0.621–1.360)	0.92 (0.621–1.358)
Crete and Islands	1.73 (1.014–2.947) *	1.81 (1.056–3.092) *
Adulteration in olive oil	0.90 (0.634–1.268)	0.86 (0.606–1.212)
Adulteration in olives	1.60 (1.156–2.220) **	1.63 (1.175–2.262) **
Olive oil production ^2^	1.34 (0.931–1.941)	1.41 (0.972–2.035)

^1^ logistic regression by profile score compared to those with a lower score. This means those that were more highly characterized by the component variables. ^2^ Olive oil production included all individuals that responded “yes” in questions about whether their family or their relatives produce olive oil (either/or). Significance at 5% level; CI, confidence interval; * *p* < 0.05; ** *p* < 0.01; *** *p* < 0.001.

## Data Availability

Data will be made available on request.

## Supplementary Materials

The following is available online at https://www.mdpi.com/article/10.3390/nu13113709/s1, Consumers’ questionnaire on knowledge, attitude, and behavior towards olive oil and table olives.
