# Supplementary material for: Attitudes towards Olive Oil Usage, Domestic Storage, and Knowledge of Quality: A Consumers’ Survey in Greece
Source: Nutrients. 2021 Oct 21;13(11):3709. doi: 10.3390/nu13113709 (PMC8621747; doi:10.3390/nu13113709)
Supplement: Supplementary file 1 [file nutrients-13-03709-s001.zip › nutrients-1421938-supplementary.pdf]

Questionnaire on olive oil consumers' knowledge, attitude and behaviour regarding choice, safety and fraudulent practices of table olives and olive oil in Greece

Date of interview: ….. / ….. / 2020

Gender: ❍ Male ❍ Female

Age group (years): ❍ 18-30 ❍ 31- 50 ❍ 51-65 ❍ >65

Place of Residence: ❍ Attica ❍ Sterea Ellada ❍ Thessaly ❍ Crete
❍ Central Macedonia ❍ East Macedonia
❍ West Macedonia ❍ Epirus ❍ Thrace
❍ Peloponnese ❍ Aegean islands ❍ Ionian islands

Number of household members (total):______ How many are below 18 years? _____

Nationality: ❍ Greek ❍ Other ……………………….

Are you actively involved in food purchasing and/or food preparation in your household?

❍ Yes ❍ No [If No, your participation in this survey ends here]

In your house, do you use olive oil? ❍ Yes ❍ No [If No, the survey ends here]

Level of education: ❍ Primary ❍ Secondary ❍ Tertiary

If university graduate: Is your degree related to food? ❍ Yes ❍ No

In your household (you can tick more than one answer)
❍ I live alone ❍ I live with my parents. No of parent/s ____
❍ I live with my partner ❍ I live with children. No of children ____

**1. Usually in your household which fat/oil do you use for;**	**Olive oil**	**Other vegetable oil** (e.g. corn oil, sunflower oil)	**Margarine**	**Butter**	**I do not know**
1.1 salads (i.e. salad dressing)	❍	❍	❍	❍	❍
1.2 preparing savoury dishes (casserole dishes, baked dishes)	❍	❍	❍	❍	❍
1.3 frying	❍	❍	❍	❍	❍
1.4 preparing pies	❍	❍	❍	❍	❍
1.5 preparing desserts (e.g. cakes, biscuits, baklava etc.)	❍	❍	❍	❍	❍

1b. Occasionally, do you use olive oil in any of the following (if not indicated in question 1)?
❍ salads
❍ preparing savoury dishes (casserole dishes, baked dishes)
❍ frying
❍ preparing pies
❍ preparing desserts (e.g. cakes, biscuits, baklava etc.)
❍ other ____

2. What type of olive oil do you use? **(you can tick more than one answer)**
❍ N/A - I do not use olive oil
❍ Extra virgin olive oil
❍ Virgin olive oil
❍ Refined oil / Olive oil composed of refined olive oils and virgin olive oils
❍ Olive pomace oil
❍ I do not know

3. Do you or your family/relatives produce olive oil?
❍ Yes ❍ No

4. Regarding the olive oil that you use: **(you can tick more than one answer)**
❍ I purchase it from retail shop (i.e. brand label from supermarket/minimarket/delicatessen shop)
❍ I purchase it "loose" in metal/tin containers from individuals that I do not know/ do not know well (i.e. not brand label)
❍ I use olive oil that is produced by me and/or my relatives and/or people that I know very well
❍ N/A - I do not use olive oil

4.1. What do you check before purchasing branded olive oil? **(Participants answer this question only if they indicate in question 4 that they purchase branded olive oil)**
❍ Packaging (container) material (e.g. glass, metal, plastic etc.)
❍ Acidity
❍ Geographical location
❍ Product of Protected Origin
❍ Health claims
❍ Price
❍ None of the above

5. An olive oil is of superior quality when: **(you can tick more than one answer):**
❍ It has high acidity
❍ It has low acidity
❍ It has bitter taste with spicy notes
❍ It is derived by cold mechanical extraction
❍ It is clear (i.e. not cloudy)
❍ It has short shelf life
❍ It is organic
❍ I do not know

6. The taste of olive oil can have bitter or spicy notes because: **(you can tick more than one answer):**
❍ It is beyond the "Best Before" date
❍ It has not been extracted properly
❍ It has high acidity
❍ It is rich in phenolic compounds with antioxidant effects
❍ I do not know

7. The statement that "*An olive oil is extra virgin when its acidity less than 0.8%*" is:
❍ Correct
❍ False
❍ I do not know

8. Between the following two types of olive oil, which one is superior with regard to its quality characteristics?
❍ Refined olive oil
❍ Extra virgin olive oil
❍ I do not know

9. Are you able to tell the difference in taste and aroma between "*refined olive oil*" and "*extra virgin olive oil*"?
❍ Yes
❍ No
❍ I do not know/ I do not answer

10. Olive oil is suitable for use as: (you can tick more than one answer)
❍ Raw in salads and dishes
❍ Cooking at low temperatures
❍ Frying at temperatures up to 190°C
❍ I do not know

11. When is olive oil best consumed?
❍ Immediately after its extraction
❍ Six months after its extraction
❍ I do not know

12. In comparison to other types of vegetable oil (e.g. sunflower oil, corn oil, rapeseed oil), do you believe that olive oil is:
❍ It is equally healthy/ nutritious
❍ Its is less healthy/ nutritious
❍ Its is more healthy/ nutritious
❍ I do not know

13. What type of containers do you have/use to keep the olive oil at home?
(you can tick more than one answer)
❍ N/A - I do not have olive oil
❍ Glass transparent containers which are not coloured
❍ Glass transparent containers which are coloured green or brown
❍ Plastic containers (as sold in retail shops)
❍ Plastic containers (initially containing water, soft drinks or other liquids and then reused to transfer olive oil from larger containers to smaller ones)
❍ Metal/tin containers
❍ Other (please specify)…………………………………………………..

14. Where do you usually keep/store the olive oil in your house?
❍ On the kitchen bench next to the oven or hotplate/stove
❍ In a cupboard (without sunlight and cool)
❍ Other
❍ I do not know/I do not remember

15. Are you concerned whether the olive oil that you use is adulterated?
❍ I am concerned
❍ I am not concerned
❍ I have never thought about it

15.1 If you are concerned, what measures have you taken or are you planning to take?
❍ I purchase/use only specific branded bottled olive oil
❍ I use only the olive oil that I and/or relatives/friends produce
❍ I do not take any measures

15.2 If you are not concerned, please justify why you are not concerned (unprompted response)
____
____
____

16. What does "*olive oil adulteration*" mean to you? (unprompted response)
____
____

❍ Mixing olive oils of different types/qualities from different parts of Greece or abroad and indicating on the label that it is extra virgin olive oil
❍ Mixing olive oil from Greece with olive oil of same type/quality from other countries and indicating on the label that the country of origin is only Greece
❍ Mixing olive oil with other vegetable oils (e.g. sunflower oil)
❍ Adding colour to vegetable oil and selling it as olive oil
❍ Having misleading label on the package (regarding its quality and place of origin)
❍ Other (please specify): ____
❍ I do not know

16b. Which of the following you would regard it as olive oil adulteration? (Prompted – indicate an answer that has not been indicated in the unprompted response)
❍ Mixing olive oils of different types/qualities from different parts of Greece or abroad and indicating on the label that it is extra virgin olive oil
❍ Mixing olive oil from Greece with olive oil of same type/quality from other countries and indicating on the label that the country of origin is only Greece
❍ Mixing olive oil with other vegetable oils (e.g. sunflower oil)
❍ Adding colour to vegetable oil and selling it as olive oil
❍ Having misleading label on the package (regarding its quality and place of origin)

17. How would you suspect olive oil fraud? My suspicion is based on: **(you can tick more than one answer)**
❍ Price
❍ Taste
❍ Colour
❍ Other. Please specify: ___________________________
❍ I do not know / I do not answer

18. Do you have a specific branded olive oil that you prefer to purchase (and you are loyal to)?
❍ Yes
❍ No
❍ I use <u>only</u> loose (non-branded) olive oil
❍ I do not use olive oil

19. Do you consume table olives?
❍ Yes
❍ No
<u>If No, please go to question 20</u>

19.1 <u>If Yes</u>, are they? **(you can tick more than one answer)**
❍ Bought prepackaged and branded
❍ Bought "loose"
❍ Prepared by myself/relatives/close friends

19.2. If you buy <u>prepackaged branded</u> table olives, do you choose a specific brand?
❍ Yes
❍ No

19.3 If you buy "loose" table olives, where do you buy them from?
❍ Supermarket
❍ Mini market / grocery store
❍ Fruit and vegetable street market (open air market)

19.4 If you buy "loose" table olives but not prepackaged branded ones, what is the reason behind this?
❍ Prepackaged branded olives have higher cost
❍ You cannot try the prepackaged olives before purchasing them
❍ Olives sold "loose" are fresher than those prepackaged
❍ Olives sold "loose" are of better quality compared to those prepackaged
❍ Other (please specify): ____
❍ I do not buy "loose" olives

20. Do you believe that table olives can be adulterated?
❍ Yes
❍ No
❍ I do not know / I have not thought about it

20.1 If Yes, how could table olives be adulterated? **(not prompted answer)**

____

____

(PROMPTED answer – you can tick more than one answer)

❍ Removing the bitterness of the olives with chemical treatment
❍ Mixing table olives of protected designation of origin with other varieties
❍ Mixing table olives from Greece with table olives from abroad
❍ Other (please specify): ____
❍ I do not know how adulteration can be done

20.2 Which of the following do you consider to be adulteration in table olives?

❍ Removing the bitterness of the olives with chemical treatment
❍ Mixing table olives of protected designation of origin with other varieties
❍ Mixing table olives from Greece with table olives from abroad
❍ Other (please specify): ____
❍ None of the above

21. How much trust do you have as consumer to the following with regard to potential olive oil adulteration? (Use of a 5-point Likert scale)

	I have no trust at all	I have no trust	I have some trust	I have trust	I have absolute trust
Olive mills	1	2	3	4	5
Olive oil distributors	1	2	3	4	5
Olive oil companies	1	2	3	4	5

22. How much trust do you have in public food safety authorities with respect to olive oil and table olives?

❍ I have no trust at all
❍ I have no trust
❍ I have some trust
❍ I have trust
❍ I have absolute trust

23. Do you think that the price of olive oil compared to other fats and oils (i.e. other vegetable oils, margarines) is:

❍ Very high
❍ High
❍ Normal
❍ Low
❍ Very low
❍ I do not know

Δ.1 What is your job? ____

Δ2. How much money do you spend per month for food purchasing (e.g. supermarkets, grocery stores)?

❍up to 50 Euros
❍51 - 100 Euros
❍101 - 150 Euros
❍151 - 200 Euros
❍201 - 250 Euros
❍251 - 300 Euros
❍301 - 350 Euros
❍351 - 400 Euros
❍401 - 450 Euros
❍451 - 500 Euros
❍≥ 501 Euros

Δ3. What is the monthly income of your household?

❍ up to 380 Euros
❍ 381 - 500 Euros
❍ 501 - 620 Euros
❍ 621 - 740 Euros
❍ 741 - 880 Euros
❍ 881 - 1.180 Euros
❍ 1.181 - 1.470 Euros
❍ 1.471 - 1.760 Euros
❍ 1.761 - 2.060 Euros
❍ 2.061 - 2.350 Euros
❍ ≥ 2.351 Euros
